# The Effect of Family Support on the Depression Levels Among Community-dwelling Older Adults in China

**DOI:** 10.1093/geroni/igaf122.3309

**Published:** 2025-12-31

**Authors:** Yuchen Gu

**Affiliations:** Boston College, Brighton, Massachusetts, United States

## Abstract

Compared to other nations, China has a severe deficit in mental health resources and support systems, particularly in professional counseling and early intervention. Current measurements used in Western samples of social support lack consideration of the unique aspects of Chinese culture, namely the focus on family support. Additionally, previous studies on family support often measure a single dimension without recognizing the complexity of its financial and emotional aspects, as well as the distinction between receiving and giving support. In this study, I categorize family support as: financial support from children (financial/receiving), living with spouse (emotional/receiving), and taking care of grandchildren (emotional/giving). Based on Chinese social and cultural backgrounds, the goal of this study is to establish how family support in Chinese distinct cultural contexts influences depression levels among community-dwelling older adults. By using the 2018 China Health and Retirement Longitudinal Study (CHARLS), I investigate the association between three different forms of family support on depression levels among Chinese older adults aged 60 and over (n = 5,187) using the regression analysis. The results show that only living with spouse and taking care of grandchildren is negatively associated with depression levels. While financial support did have a negative impact, it is not statistically significant. These results show that emotional and psychological support are more important in the prevention of mental health deterioration for Chinese older adults. The research findings suggest that family support for Chinese older adults should go beyond financial help and focus more on emotional aspects.

